# Supplementary material for: Three ParA Dimers Cooperatively Assemble on Type Ia Partition Promoters
Source: Genes (Basel). 2021 Aug 28;12(9):1345. doi: 10.3390/genes12091345 (PMC8465637; doi:10.3390/genes12091345)
Supplement: Supplementary file 1 [file genes-12-01345-s001.zip › genes-1327013-supplementary.pdf]

Supplementary data

Three ParA dimers cooperatively assemble on type Ia partition promoters

François Boudsocq[1], Maya Salhi[1] Sophie Barbe[2] and Jean-Yves Bouet[1]

[1] Laboratoire de Microbiologie et Génétique Moléculaires, Centre de Biologie Intégrative (CBI), Centre National de la Recherche Scientifique (CNRS), Université de Toulouse, UPS, F-31062 Toulouse, France.

[2] Toulouse Biotechnology Institute (TBI), Université de Toulouse, CNRS, INRAE, INSA, F-31077 Toulouse, France.

The supplementary data includes:

Figure S1. ParA$_F$ purification steps and specific DNA binding activity. 2

Figure S2. Oligonucleotides used in this study. 3

Figure S3. Weak DNA binding of ParA$_F$ on DNA fragment smaller than 80-bp and non-specific DNA binding of ParB$_F$. 4

Figure S4. Secondary structure analysis of ParA$_F$. 5

Figure S5. *P*parAB$_F$ homologous sequence. 6

Figure S6. ParA$_F$ and ParA$_{P1}$ dimers undergo large conformation changes in the wing domain. 7

Table S1. Summary of the binding and kinetic parameters determined by SPR analyses. 8

Figure S1. ParA$_F$ purification steps and specific DNA binding activity.

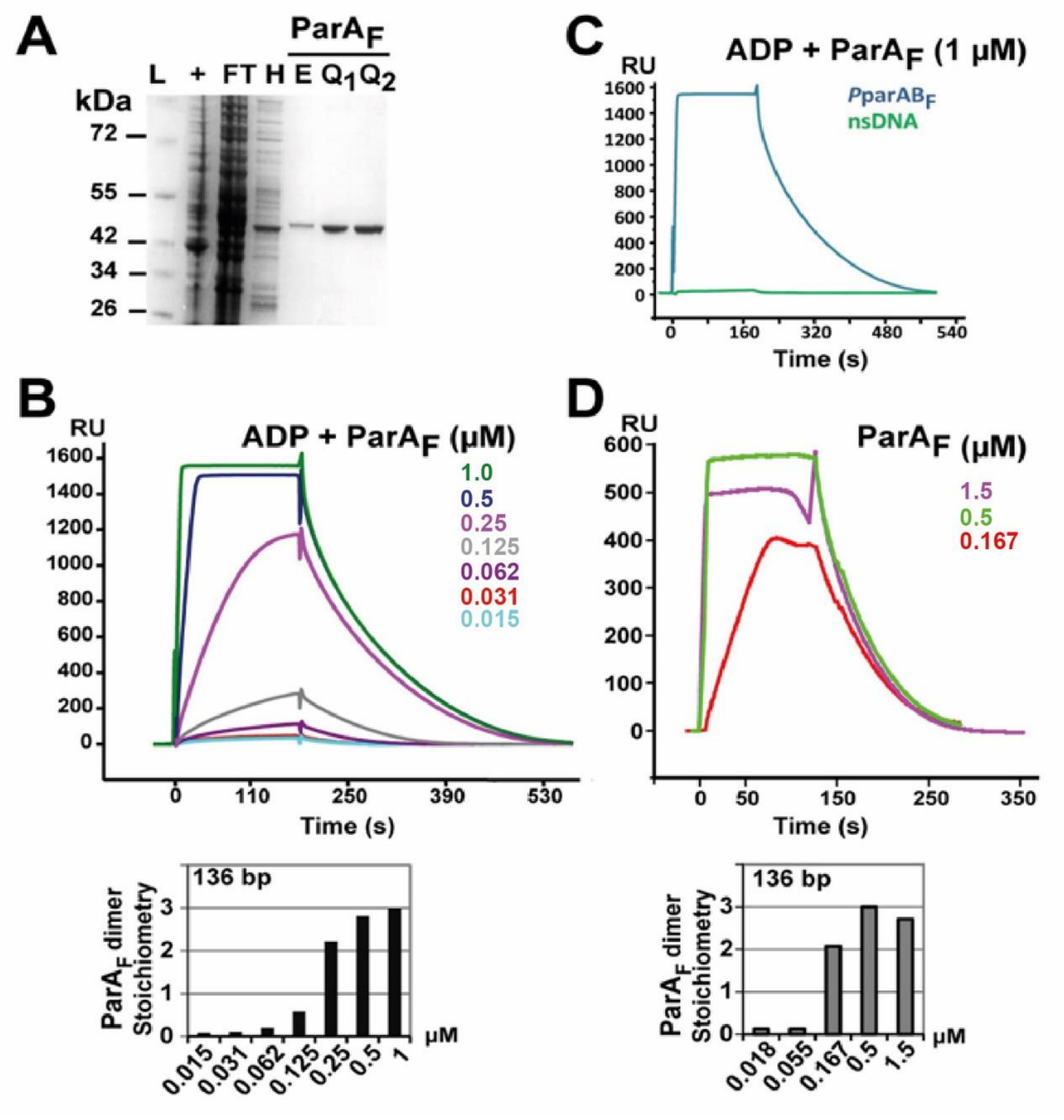

A. Purification steps of ParA$_F$. The protein fractions during the purification procedure were analyzed on a 4-20% SDS-PAGE and colored with Instant Blue (Euromedex). Samples were crude extract from induced culture (+), flow through of the heparin column (FT), heparin fraction (H), exclusion fraction of Superdex 200 (E), monoQ fractions (Q1-Q2). Molecular weight ladder (L) is indicated on the left in kDa.

B. SPR analysis of ParA$_F$ binding to a 136-bp *P*parAB$_F$ DNA fragments in presence of 1 mM ADP. Upper panel: 530 RU of biotinylated DNA was immobilized on streptavidin SA chip (Biacore 3000). Increasing concentration of ParA$_F$ (from 0.015 to 1 µM) was injected (time 0) at 10 µL/min for 180 s before dissociation. Lower panel: stoichiometry of ParA$_F$ dimers bound per *P*parAB$_F$ DNA fragment, calculated as in Figure 2B. Three ParA$_F$ dimers are bound per DNA fragment at saturation.

C. ParA$_F$ binds specifically to the promoter region in the presence of ADP. SPR analysis (Biacore 3000) of ParA$_F$ binding to a 136-bp DNA fragments containing *P*parAB$_F$ (blue) or nsDNA (green) performed as in Figure 2B except for the presence of 1 mM ADP in the reaction.

D. SPR analysis as in (B) using a Biacore X100 with 200 RU of 136-bp *P*parAB$_F$ fragment immobilized. ParA$_F$ proteins (from 0.018 to 1.5µM) were injected at 30 µL/min. The three higher concentrations, 0.17, 0.5 and 1.5 µM are displayed in red, green and purple, respectively. Lower panel: stoichiometry of ParA$_F$ dimers bound per *P*parAB$_F$ DNA fragment, calculated as in Figure 2B. Three ParA$_F$ dimers are bound per DNA at saturation without ADP.

Figure S2. Oligonucleotides used in this study.

TTGTTGTTGCTTTGCAGTAAATTGCAAGATTTAATAAAAAAAACGCAAAGCAATGATTA	58bp	34	btn
GGTTTTTTGATGAGTCTCTGATTTTGTTGTTGCTTTGCAGTAAATTCAGAGATTTAATAAAAAAAACGCAAAGCAATGATT	80bp	34	cy3
TGACTCGTCGACCTGATTATTAGTCTGGGACCACGGTCCCATGCTTTGCAGTAAATTGTCAGATTTAATAAAAAAAACGCAAAGCA	85bp	34	btn
TTAAACAAGTCTCTGGTTTTTTTGATGACTTTGCGATTTTGTTGTTGCTTTGCAGTAAATTGCAAGATTTAATAAAAAAAACGCAAAGCAATGATTAAAGGA	100bp	22′34	cy3
GTAGTGTTGCTCTTATTTTAAACAACTTTGCGGTTTTTTTGATGACTTTGCGATTTTGTTGTTGCTTTGCAGTAAATTGCAAGATTTAATAAAAAAAACGCAAAGCAATGATTAAAGGATGT	120bp	122′34	cy3
ATCTCCTTTTGTAGTGTTGCTCTTATTTTAAACAACTTTGCGGTTTTTTTGATGACTTTGCGATTTTGTTGTTGCTTTGCAGTAAATTGCAAGATTTAATAAAAAAAACGCAAAGCAATGATTAAAGGATGTTCAGAA	136bp	122′34 $PparAB_F$	btn
CAGTGAATTGTAATACGACTCACTATAGGGCGAATTGGAGCTCCACCGCGGTGGCGGCCGCTCTAGAACTAGTGGATCCCCCGGGCTGCAGGAATTCGATATCAAGCTTATCGATACCGTCGACCTCGAGGGGGGG	136bp	ns DNA	btn
GTAGTGTTGCTCTTATTTTAAACAACTTTGCGGTTTTTTTGATGACTTTGCGATTTTGTTGTTGTCAGTAAGTAAATTAGTCATTTTAATAAAAAAAACTCAGTACAATGATTAAAGGATGT	120bp	12	cy5
GTAGTGTTGCTCTTATTTTAAACAACTTTGCGGTTTTTTTGATGATCAGTAGATTTTGTTGTTGTCAGTAAGTAAATTAGTCATTTTAATAAAAAAAACGCAAAGCAATGATTAAAGGATGT	120bp	14	cy5
GTAGTGTTGCTCTTATTTTAAACAACTTTGCGGTTTTTTTGATGACTTTGCGATTTTGTTGTTGCTTTGCAGTAAATTAGTCATTTTAATAAAAAAAACTCAGTACAATGATTAAAGGATGT	120bp	123	cy5
GTAGTGTTGCTCTTATTTTAAACAATCAGTAGGTTTTTTTGATGATCAGTAGATTTTGTTGTTGCTTTGCAGTAAATTAGTCATTTTAATAAAAAAAACGCAAAGCAATGATTAAAGGATGT	120bp	34	cy5
GTAGTGTTGCTCTTATTTTAAACAATCAGTAGGTTTTTTTGATGACTTTGCGATTTTGTTGTTGCTTTGCAGTAAATTAGTCATTTTAATAAAAAAAACGCAAAGCAATGATTAAAGGATGT	120bp	234	cy5
GTAGTGTTGCTCTTATTTTAAACAATCAGTAGGTTTTTTTGATGATCAGTAGATTTTGTTGTTGCTTTGCAGTAAATTGCAAAGTTTAATAAAAAAAACGCAAAGCAATGATTAAAGGATGT	120bp	32*4	cy5
GTAGTGTTGCTCTTATTTTAAACAATCAGTAGGTTTTTTTGATGACTTTGCGATTTTGTTGTTGTCAGTAAGTAAATTGCAAAGTTTAATAAAAAAAACTCAGTACAATGATTAAAGGATGT	120bp	22*	cy5
GTAGTGTTGCTCTTATTTTAAACAATCAGTAGGTTTTTTTGATGACTTTGCGATTTTGTTGTTGTCAGTAAGTAAATTGCAAAGTTTAATAAAAAAAACGCAAAGCAATGATTAAAGGATGT	120bp	22*4	cy5

The indicated oligonucleotides are annealed with their complementary oligonucleotides to generate duplex DNA probes. Note that each DNA fragment is designated by its length in base pair (bp). The binding motifs are labelled with the same color code as in Figure 5C, and are indicated by their numbering at the end of the line. Cy3 or biotin (btn) modifications at the 5’ extremities of the indicated oligonucleotides are also listed.

Figure S3. Weak DNA binding of ParA$_F$ on DNA fragment smaller than 80-bp and non-specific DNA binding of ParB$_F$.

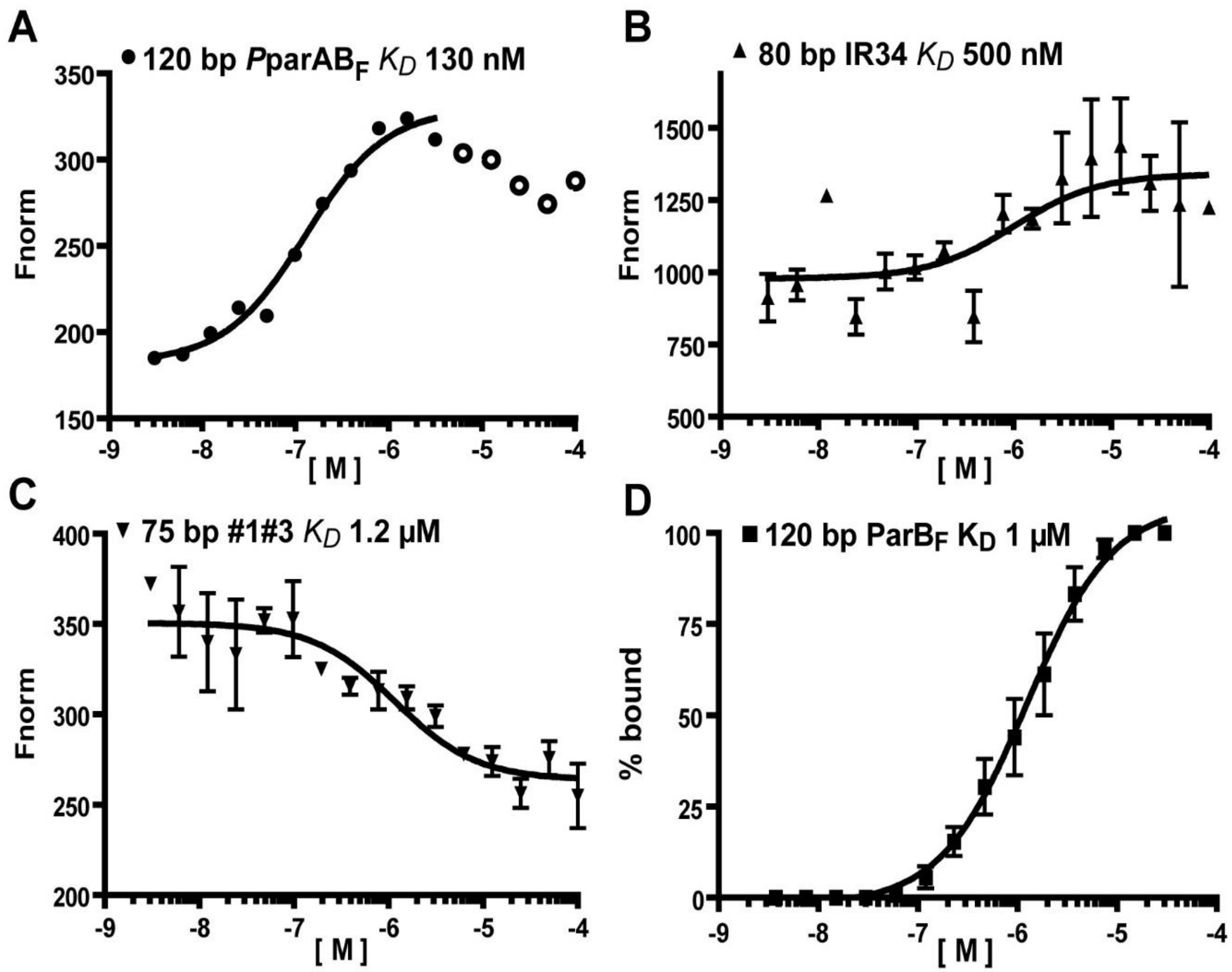

Quantification of the DNA binding affinities of ParA$_F$ (A-C) and ParB$_F$ (D) to various DNA substrates measured by fluorescence (A-C) or EMSA (D). The resulting curves were fitted using non-linear regression by Prism to calculate an apparent K_D. Experiments have been reproduced three times except for (A).

A. A 120-bp Cy3 labelled DNA probe (25 nM) containing *P*parAB$_F$ was titrated with 3.6 nM to 100 µM ParA$_F$. The fluorescence measurements lead to an apparent K_D of 130 nM. Decreasing signals above 2µM was due to aggregation, detected by denaturation measurement within the assays.

B. A 80-bp Cy3 labelled DNA probe (15 nM) containing IR3-4 was titrated with 3.6 nM to 100 µM ParA$_F$. The fluorescence measurements lead to an apparent K_D of 500 ± 325 nM.

C. A 75-bp Cy3 labelled DNA probe (15 nM) containing the binding motifs #1 and #3 was titrated with 3.6 nM to 100 µM ParA$_F$. The fluorescence measurements lead to an apparent K_D of 1200 ± 700 nM.

D. A 120-bp Cy3 labelled DNA probe (15 nM) was titrated with 3.7 nM to 30 µM ParB$_F$ and analyzed in EMSA. The quantification of the free versus retarded DNA leads to an apparent K_D of 1 ± 0.2 µM, in agreement with previous measurements [1].

Figure S4. Secondary structure analysis of $ParA_F$.

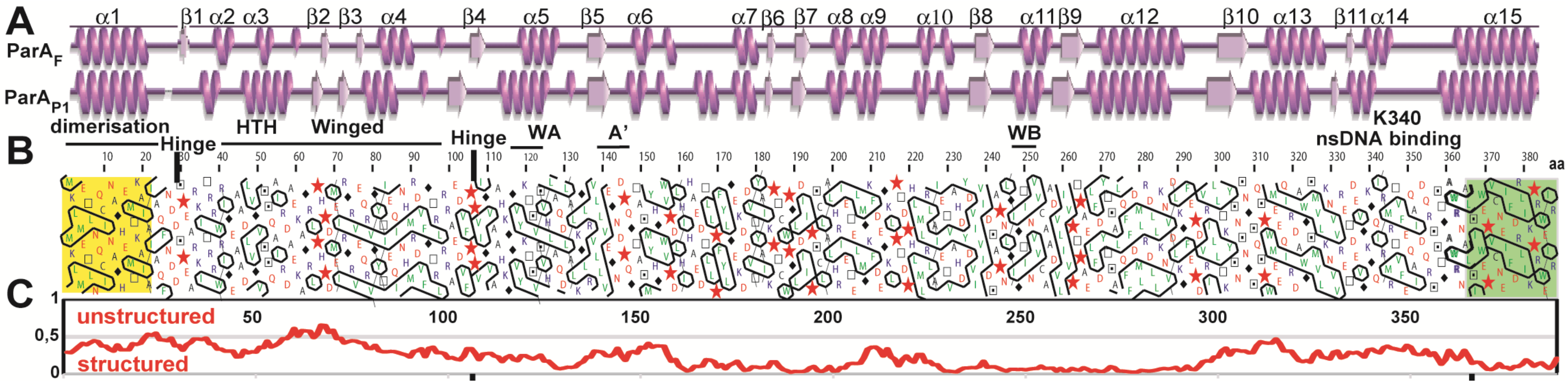

A. Comparison of the secondary structures of $ParA_F$ (top) and $ParA_{P1}$ (bottom). Secondary structures were generated by PDBsum from the 3-D model of $ParA_F$ and the X-ray structure of ADP $ParA_{P1}$ (3ez2). The α-helices (solenoids) and β-strands (arrows) are numbered according to $ParA_F$.

B. Hydrophobic cluster analysis plot of $ParA_F$ protein [2] The numbering of amino acids residues is labelled on top. Key amino acids are highlighted by symbols as follows: proline (star), glycine (diamond), threonine (empty square) and serine (dotted square). The different domains and motifs are labelled as in Figure 4A. The first and the last α-helices are colored in yellow and green, respectively.

C. Analysis of intrinsically unstructured/disordered domains of $ParA_F$ protein performed using IUPred [3].

Figure S5. *P*parAB$_F$ homologous sequence.

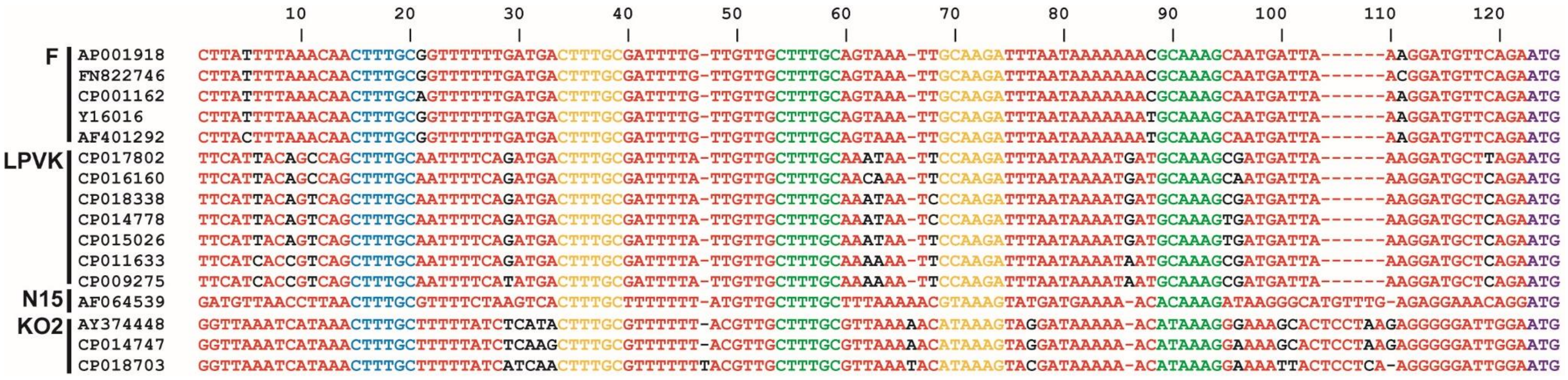

Homologous sequences of *P*parAB$_F$, *P*parAB$_{LPVK}$, *P*parAB$_{N15}$, and *P*parAB$_{KO2}$ were searched by Blastn analyses. Only the sequences presenting at least one nucleotide difference are displayed. The first sequence of each group (indicated on the left) corresponds to the promoter used for the Blastn search, with the nucleotide variation within a group labelled in black. Sequences closely related to *P*parAB$_F$ are: AP001918 (*E. coli* K-12 plasmid F), FN822746 (*E. coli* ETEC 1392/75 plasmid p557), CP001162 (*E. coli* Vir68 plasmid pVir68), Y16016 (*E. coli* plasmid pO157), AF401292 (*E. coli* O157:H- plasmid pSFO157).

Sequences closely related to *P*parAB$_{LPVK}$ are: CP017802 (*Raoultella ornithinolytica*), CP016160 (*Klebsiella pneumoniae*), CP018338 (*K. pneumoniae* isolate Kp_Goe_154414 plasmid pKp_Goe_414-2), CP014778 (*Pluralibacter gergoviae* strain FB2 plasmid pFB2.3), CP015026 (*K. pneumoniae* strain Kpn223 plasmid pKPN-065), CP011633 (*Klebsiella oxytoca* strain CAV1374 plasmid pCAV1374-150), CP009275 (*K. variicola* strain DX120E plasmid pKV1). No closely related sequence to *P*parAB$_{N15}$ (AF064539) was found. Sequences closely related to *P*parAB$_{KO2}$ are: AY374448 (*Bacteriophage phiKO2*), CP014747 (*Enterobacter aerogenes* strain FDAARGOS_139 plasmid), CP018703 (*K. pneumoniae* strain Kp_Goe_827024 plasmid pKp_Goe_024-5). ParA$_F$ binding motifs are colored as in Figure 5C. The ATG start codons at the end of the sequence are colored in purple.

Figure S6. ParA$_F$ and ParA$_{P1}$ dimers undergo large conformation changes in the wing domain.

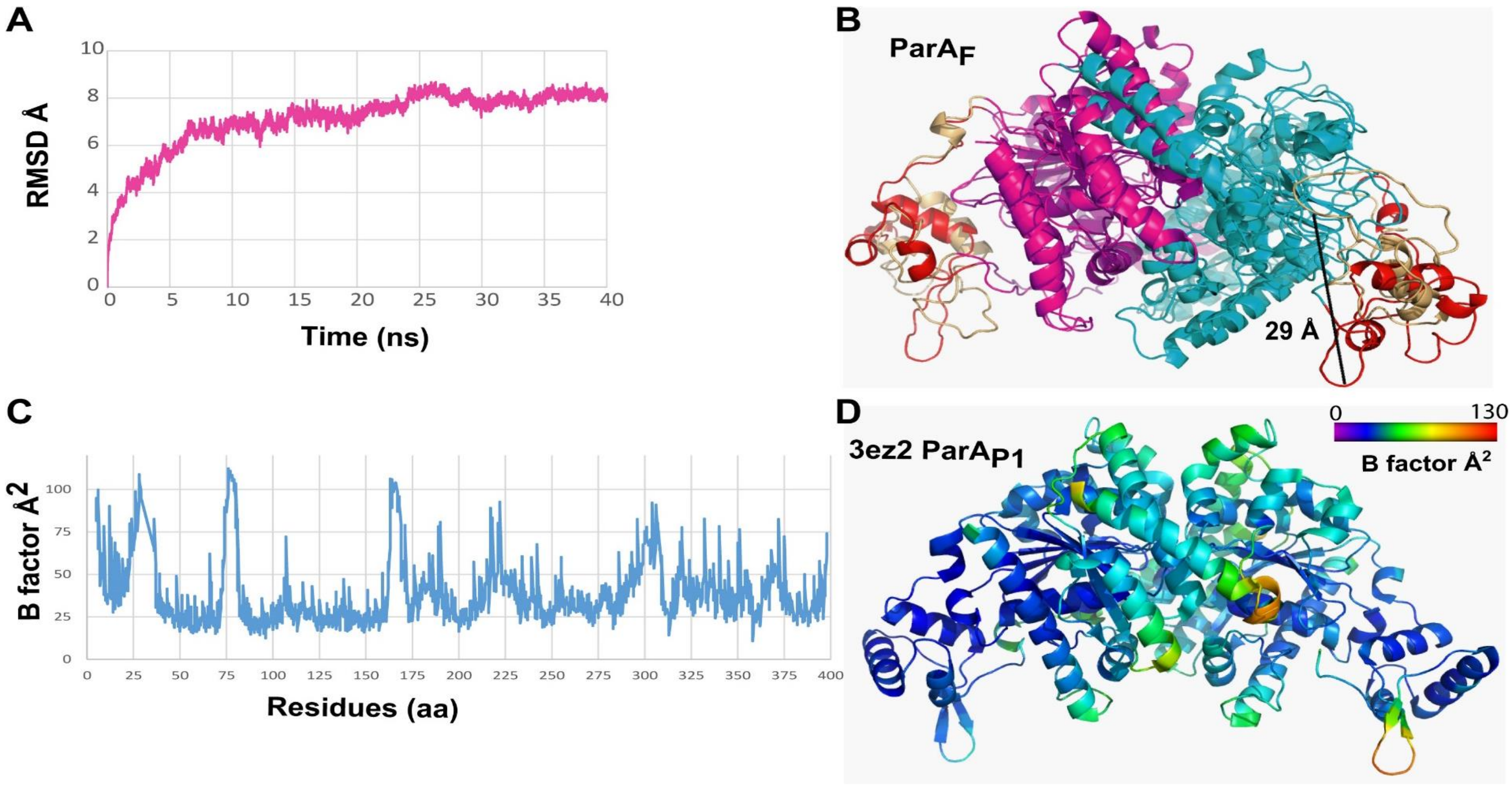

A. Quantitative measurement of ParA$_F$ movements during MD simulations. Root mean squared deviation of ParA$_F$ backbone atoms of ParA$_F$ monomer is displayed as a function of time during 40 ns simulation of in explicit water. **B**. Backbone superposition of the starting model of ParA$_F$ dimer at 0 ns and the snapshot after 40 ns MD simulation (monomers are colored pink and cyan). Winged-HTH region of two states (0 and 40 ns) are colored in red and salmon, respectively. Distance between the arginine 75 C-alpha atoms in initial and 40 ns structure is shown in black. **C**. Plot of the ParA$_{P1}$ residues B-factors reported in the 3ez2 PDB files [4]. **D.** Schematics of ParA$_{P1}$ X-ray structure. The fold is shown in cartoon and colored-coded according to B-factors plotted in (C).

Table S1. Summary of the binding and kinetic parameters determined by SPR analyses.

K_D	*ka* $M^{-1} s^{-1}$	*kd* s^{-1}	Chi^2	R_{max}	RU immobilized	Flow µL/min	Apparatus
472 nM[a]	1.9 10^4	0,0092	490	2300	530	10	Biacore3000
640 nM[b]	1,39 10^4	0,0089	220	2000	530	10	Biacore3000
450 nM[c]	6,89 10^4	0,0318	880	850	300	20	Biacore3000
70 nM[d]	3,87 10^5	0,0274	1000	650	200	30	BiacoreX100

The binding and kinetics parameters from the SPR analyses assaying the $ParA_F$-*P*parABS_F interaction described in the main text and Supplementary materials are summarized along with the experimental conditions and apparatus used for each analysis. All experiments were performed on a 136-bp DNA fragments containing *P*parAB_F region. Note that the Chi^2 values for the experiments performed with conditions [c] and [d] are close to the Rmax values indicating that the accuracy of these experiments is low. As mentioned in the main text, these variations depend mainly on the tendency of $ParA_F$ to self-aggregate and therefore the kinetics parameters have to be taken as an indication of the interaction.

[a] Data sets in which $ParA_F$ concentrations vary from 0.015 to 1 µM.

[b] Data sets performed in the presence of 1 mM ADP from Figure S1B.

[c] Data sets from Figure 2A.

[d] Data sets from Supplementary Figure S1D.

Supplementary References

1. Ah-Seng, Y., Lopez, F., Pasta, F., Lane, D., and Bouet, J.Y. (2009). Dual role of DNA in regulating ATP hydrolysis by the SopA partition protein. J Biol Chem *284*, 30067-30075.
2. Callebaut, I., Courvalin, J.C., Worman, H.J., and Mornon, J.P. (1997). Hydrophobic cluster analysis reveals a third chromodomain in the Tetrahymena Pdd1p protein of the chromo superfamily. Biochem Biophys Res Commun *235*, 103-107.
3. Dosztanyi, Z., Csizmok, V., Tompa, P., and Simon, I. (2005). IUPred: web server for the prediction of intrinsically unstructured regions of proteins based on estimated energy content. Bioinformatics *21*, 3433-3434
4. Dunham, T.D., Xu, W., Funnell, B.E., and Schumacher, M.A. (2009). Structural basis for ADP-mediated transcriptional regulation by P1 and P7 ParA. Embo J *28*, 1792-1802
